# Synergistic effect of carbomer polymer and graphene oxide nanoparticles on the rat wound healing process

**DOI:** 10.22038/ijbms.2025.89073.19227

**Published:** 2025

**Authors:** Yousef Moradian, Tahereh Sadat Tabatabai, Tayebeh Sadat Tabatabai, Amir Atashi, Vahid Shirshahi

**Affiliations:** 1 Student Research Committee, School of Medicine, Shahroud University of Medical Sciences, Shahroud, Iran; 2 Department of Basic Sciences, School of Medicine, Shahroud University of Medical Sciences, Shahroud, Iran

**Keywords:** Graphene, Hydrogels, Polymers, Tissue Regeneration, Wound Healing

## Abstract

**Objective(s):**

Wound healing is a multifaceted process involving inflammation, proliferation, and remodeling phases. Effective wound management requires materials that can promote tissue regeneration while preventing infections. Carbomer polymers are widely used in wound dressings due to their biocompatibility and water absorption properties, while graphene oxide nanoparticles have shown potential in enhancing cellular activities and antimicrobial effects. However, the combined effect of these materials on wound healing remains unexplored.

**Materials and Methods:**

A composite hydrogel was prepared by incorporating graphene oxide nanoparticles into a carbomer polymer matrix. The hydrogel’s physicochemical properties, including swelling behavior, mechanical strength, and drug release profile, were evaluated. *In vivo *wound healing studies were conducted on Sprague-Dawley rat models, with wounds treated using the composite hydrogel and a control group.

**Results:**

The composite hydrogel demonstrated superior swelling capacity and mechanical stability. *In vivo* studies revealed significantly faster wound closure rates in the composite hydrogel-treated group (*P*<0.05). Histological analysis showed enhanced tissue regeneration, reduced inflammation, and increased collagen deposition in the treated wounds.

**Conclusion:**

The synergistic combination of carbomer polymer and graphene oxide nanoparticles effectively enhances wound healing by improving hydrogel properties and promoting tissue regeneration. This composite material holds significant potential for advanced wound care applications.

## Introduction

Approximately 15% of the body’s weight is composed of skin, which is the largest organ in the body (1, 2). It serves multiple vital functions, including protecting the body from environmental elements and invading pathogens. Any damage to the skin disrupts its natural structure and function, leading to wound formation (3), which is generally classified into acute and chronic. Unlike chronic wounds, which are difficult to heal and require restoration of the normal anatomical structure and function, acute wounds tend to heal more quickly and spontaneously. Healing of damaged tissue is a complex process that can be addressed using various techniques, depending on the type and severity of the wound. These methods include the application of skin grafts, stem cells, and their derivatives to regenerate lost tissue. Despite the advancements, no current method provides a perfect or complete solution for wound healing, highlighting the ongoing need for the development of improved treatment strategies (4).

Graphene is a two-dimensional nanomaterial composed entirely of carbon atoms arranged in a hexagonal lattice. It exhibits outstanding electrical and thermal conductivity, exceptional tensile strength, high flexibility, and optical transparency, making it one of the most durable and versatile nanomaterials. Graphene oxide (GO) is a derivative of graphene, characterized by oxygen-containing groups on its surface, which enhance its dispersibility in water and other solvents, while maintaining many of graphene’s beneficial properties (5-8). 

Research indicates that graphene and its derivatives can induce the production of intracellular nitrogen and reactive oxygen species (ROS), which play a crucial role in promoting angiogenesis. ROS can phosphorylate protein kinase B (Akt), thereby increasing the activity of endothelial nitric oxide synthase (eNOS). Elevated ROS levels enhance eNOS activity, which activates nitric oxide (NO) signaling pathways. Increased intracellular NO further facilitates angiogenesis, supporting processes such as wound healing (9). Thanks to its distinctive properties, graphene exhibits potent antibacterial activity against both gram-positive and gram-negative bacteria (10, 11). Studies have demonstrated that GO can effectively modify the shape and orientation of mesenchymal stem cells without hindering their normal growth. Its two-dimensional structure and smooth, uniform surface create an environment conducive to cell adhesion, primarily through van der Waals and electrostatic interactions, which promote cell attachment and proliferation (12). Moreover, graphene’s mechanical properties are exceptional; it can be bent and revert to its original shape under pressure or stress. This flexibility allows graphene to adapt to various biological environments and support cell survival, even in distorted conditions (13).

Acrylic acid polymers, known as carbomers, are cross-linked, high molecular weight polymers that are typically available as dry, lightweight powders (14, 15). These polymers are widely used across various industries, including pharmaceuticals and cosmetics, where they serve as excipients in gels, creams, and other formulations. Due to their high content of carboxylic groups (56–58%) in their structure, they form porous, elastic networks capable of retaining water. This enhanced swelling capability plays a significant role in wound healing by promoting cell survival and growth during the healing process. Additionally, carbomers can function as carriers, delivering nutrients or medications to cells while maintaining a moist and conducive environment that supports cellular health (16, 17). Other important properties include high viscosity, excellent heat stability, good biocompatibility with tissues, and compatibility with a wide range of drugs (17-20).

Given the significance of GO nanoparticles and carbomer 980 in wound healing and angiogenesis, this study aims to investigate the synergistic effects of carbomer 980 and graphene in promoting efficient wound repair.

## Materials and Methods

### Materials

To ensure the consistency and reliability of the experimental results, all primary materials were sourced from reputable suppliers. Sulfuric acid (H₂SO₄) and hydrochloric acid (HCl) were purchased from Merck in Germany. Ammonia, hydrogen peroxide (H₂O₂), potassium permanganate (KMnO₄), and graphite powder—the precursor for GO synthesis—were obtained from Acros Organics in Belgium, ensuring high purity for optimal outcomes. Deionized water was used as the solvent at every stage of synthesis and preparation to minimize contamination. For biological evaluations, mouse fibroblast cells (3T3), a standard model for biocompatibility studies, were acquired from the Pasteur Institute of Iran. Additionally, the reagent 3-(4,5-dimethylthiazol-2-yl)-2,5-diphenyltetrazolium bromide (MTT) was purchased for cytotoxicity assessments to evaluate the impact of the nanofiber scaffolds on cell viability. Pharmaceutical-grade carbomer 980 was supplied by Sigma-Aldrich (Merck, Germany).

### Synthesis of GO NPs

A modified Hummer’s method was used to synthesize GO. Initially, 5.75 ml of 98% H_2_SO_4_ was combined with 0.2 g of graphite powder and magnetically stirred for three hours. Subsequently, 0.75 g of KMnO₄ was gradually added to the mixture in an ice-cold water bath. After 30 min of stirring, the temperature was raised to 40 °C. The mixture was then stirred at 70 °C for an additional 15 min, resulting in a dark brown slurry. Next, 0.75 ml of distilled water was added, and the mixture was heated to 105 °C for five minutes. An additional 0.75 ml of distilled water was then introduced, maintaining the temperature at 105 °C for another five minutes. The mixture was further diluted with 10 ml of distilled water and kept at 100 °C for 15 min. To complete the oxidation process, 2.5 ml of 30% H₂O₂ and 35 ml of distilled water were added. The mixture was then centrifuged at 9000 g for 12 min at room temperature.

Following centrifugation, the supernatant was discarded, and the precipitate was washed twice with 5% HCl to remove impurities. Finally, the particles were rinsed thoroughly with distilled water until the pH was neutral, ensuring removal of residual acids.

### Hydrogel preparation

Initially, a 1% (w/v) solution of carbomer 980 was prepared using distilled water. This solution was then mixed with another solution containing 0.05% (w/v) GO nanoparticles. To achieve a uniform dispersion of the GO nanoparticles, the mixture underwent ultrasonic treatment for ten minutes. Finally, the hydrogel was formed by homogenizing the mixture for an additional ten minutes.

### Morphology and structure observation

Scanning electron microscopy (SEM) was utilized to analyze the morphology and structure of the materials (Hitachi, Japan). After applying a sputter coating, the samples were placed on holders and inserted into the SEM chamber. Digital images were captured, and ImageJ software was employed to measure the pore sizes and calculate the average pore size.

### FTIR spectra measurements

Fourier transform infrared spectroscopy (FTIR) was performed in reflection mode within the mid-infrared range of 400 to 4,000 cm⁻¹. This technique was used to identify specific chemical and functional groups present in the scaffolds. For the analysis, 300 mg of infrared-grade potassium bromide (KBr) was carefully mixed with 1 mg of the powder sample, and the mixture was compressed into pellets under vacuum. The pellets were then analyzed with a resolution of 4 cm⁻¹ and a scanning speed of 120 scans per minute.

### Cell viability

Cell viability was evaluated colorimetrically using the MTT assay, which relies on the ability of metabolically active mitochondria in living cells to convert the yellow tetrazolium salt MTT into purple formazan crystals. The viability of 3T3 murine fibroblast cells cultured on the hydrogels was assessed using the MTT test kit. Cells were seeded onto the hydrogels at a density of 1 × 10² cells and incubated for 1 and 3 days in a humidified incubator at 37 °C with 5% CO₂, using DMEM/F12 culture medium supplemented with 10% (v/v) fetal bovine serum (FBS), 100 units/ml penicillin, and 100 μg/ml streptomycin. After incubation, 150 μl of MTT solution (0.5 mg/ml) was added to the culture medium and incubated for four hours at 37 °C in the dark. The resulting formazan crystals were dissolved by adding 100 μl of DMSO, and the absorbance of the solution was measured at 540 nm using a microplate reader (Anthos 2020, Biochrom, Berlin, Germany). Finally, cell viability was calculated using the following formula.

Cell Viability (%)= OD Sample/ OD Positive Control × 100

### Anti-inflammatory test

The anti-inflammatory test is used to evaluate tissue damage and protein structural changes *in vitro *by measuring a substance’s ability to inhibit or lessen inflammatory responses. In this procedure, 0.1 grams of the sample is mixed with 2.8 milliliters of phosphate-buffered saline (PBS) at pH 6.8 and 0.2 milliliters of 1% albumin. The mixture is incubated at 37 °C for 15 min, then heated at 70 °C for five minutes. Finally, the absorbance is recorded at 660 nm. In this assay, the combination of PBS and 1% albumin serves as the control, and aspirin is used as the standard reference.

Protein denaturarion (%) = Acontrol - Asample / Acontrol × 100

### Lactate dehydrogenase test

The LDH (Lactate Dehydrogenase) assay is a chemical test used to assess the extent of cell damage and death. Since all cells contain the cytosolic enzyme LDH, which plays a role in energy metabolism, the amount of LDH released into the cell culture medium serves as an indicator of cellular injury—this enzyme leaks out when the cell membrane is compromised. In this study, cell viability was evaluated using the LDH assay. The amount of released LDH enzyme was measured with an LDH detection kit (Sigma-Aldrich), and the optical density was recorded at 490 nm using an ELISA reader.

### Blood compatibility test

The Blood Compatibility (BC) test is conducted to evaluate the safety of materials that come into contact with blood, specifically to determine whether they cause adverse effects such as blood clotting. For this test, 2 ml of fresh anticoagulated human blood was diluted with 2.5 ml of normal saline. Then, 0.2 ml of this diluted blood was mixed with the sample. After incubating the mixture at 37 °C for 60 min, it was centrifuged at 1500 rpm for ten minutes. The resulting supernatant was transferred to a 96-well plate, and absorbance was measured at 545 nm using an Anthos 2020 microplate reader (Biochrom, Berlin, Germany). As controls, 0.2 ml of diluted blood in 10 ml of deionized water served as the positive control, while 0.2 ml of diluted blood in 10 ml of normal saline was used as the negative control. Finally, hemolysis percentage was calculated using the following formula.

Hemolysis (%) = Dt - Dnc/Dpc - Dnc × 100

Where Dt shows the optical density of the sample, Dnc indicates the optical density of the negative control, and Dpc is the absorbance of the positive control.

### Blood clotting index

The Blood Clotting Index (BCI) test is a method used to evaluate a substance’s ability to affect blood clotting time, either by accelerating or delaying it. To perform the test, approximately 0.1 grams of each sample was placed into a 25 ml beaker and incubated in a water bath at 37 °C for one hour. After five minutes, 20 μl of 0.2 M CaCl₂ was added to the hydrogels, followed by 100 μl of anticoagulated blood. Another five minutes later, 25 ml of double-distilled water (ddH₂O) was slowly added. As a control, ddH₂O containing anticoagulated blood was used. Finally, the absorbance of 100 μl of the supernatant was measured at 545 nm. The results were then calculated using the following formula.

Blood Clotting Index (%) = A sample / A control × 100

The amount of sample absorption is represented by A Sample, whereas A Control stands for the control absorption value.

### Blood uptake test

This test evaluates the amount of blood absorbed by a material or surface. It is commonly performed to study the distribution and absorption of various substances within the circulatory system. Specifically, the sample intended to contact blood is immersed in whole human blood for set time intervals, such as every ten and twenty minutes at room temperature. After measuring the initial weight (W0) and the wet weight (W1) post-immersion, the blood absorption capacity is calculated using the following formula.

Blood uptake capacity (%) = W1 - W0 / W1 × 100

### pH assessment

The pH test is a simple and widely used method to determine the acidity or alkalinity of a solution by measuring the concentration of hydrogen ions (H+). It can also monitor how the pH changes over time. In this study, samples of equal weight were immersed in simulated bodily fluid (SBF) at 37 °C with an initial pH of 7.4. The pH of the solution was measured at several time points—2, 4, 6, 12, and 72 hr—using a Mettler Toledo pH meter (Greifensee, Switzerland).

### Total anti-oxidant capacity

The total anti-oxidant capacity index of biological samples or skin measures the tissue’s ability to neutralize free radicals and ROS. This test evaluates how well the skin can defend itself against oxidative damage, which is linked to premature aging, inflammation, and UV-related harm. In studies involving skincare, cosmetics, and skin disorders, a higher anti-oxidant capacity suggests that the skin is more capable of defending cells from oxidative stress. In this study, an extract from skin tissue cells is prepared and combined with the anti-oxidant reagent ABTS. The resulting solution’s color change is then measured at 734 nm using a spectrophotometer. This measurement helps determine the anti-oxidant potential of the sample.

### Weight loss test

The weight loss test is a scientific method used to evaluate the biostability and degradation of materials, especially scaffolds and biodegradable substances. In this procedure, samples of the materials are precisely weighed and placed in simulated environments, such as physiological conditions or controlled habitats with specific pH and temperature. At designated time points, the samples are removed from the medium, gently washed with PBS or deionized water to eliminate any residues, and then dried at an appropriate temperature (commonly 37 °C) until their weight stabilizes. The percentage of weight loss is then calculated using a formula. In this study, each lyophilized scaffold was initially weighed (W0), submerged in 20 ml of PBS solution (pH 7.4) at room temperature for two and four hours, then washed with deionized water, frozen, lyophilized again, and weighed (W1) after each time interval. The following equation was used to calculate the percentage weight loss of the hydrogels.

Weight Loss (%) = W0 W1 / W1 × 100

### Water absorption test

Water absorption testing is a key method for assessing the hydrophilic characteristics of various materials such as polymers, hydrogels, and other biomaterials. In this test, samples with specific dimensions (like films, matrices, or polymer pieces) are immersed in water, and their weight is recorded after a certain time period. The percentage increase in weight is calculated to indicate the amount of water absorbed. To evaluate the swelling behavior of hydrogels, the dried scaffolds were first weighed (Wd) and then immersed in PBS solution (pH 7.4) at 37 °C. At set time points, the swollen samples were removed, surface moisture was gently blotted off with filter paper, and the samples were weighed again (Ws). Each experiment was performed in triplicate. The percentage swelling of the hydrogels was calculated using the following formula.

S(%) = (Ws - Wd) / Wd × 100

### DAPI test

The DAPI (4′,6-diamidino-2-phenylindole) assay is a staining method used to visualize cell nuclei and assess nuclear changes, particularly in studies of genetic damage, cell division, and cell death. DAPI is a fluorescent dye that binds specifically to DNA by attaching non-selectively to purine bases, emitting fluorescence visible under a fluorescence microscope. To evaluate cell attachment to the scaffolds, nuclear staining with DAPI was performed. After 24 hr of cell seeding, the scaffolds were fixed by immersing them in 4% paraformaldehyde for eight minutes. Then, to enhance dye penetration, samples were treated with 0.1% Triton X-100 for ten minutes, followed by three washes with PBS. Next, 50 μl of DAPI staining solution at room temperature was added to each well. After five minutes, the dye was removed, and the scaffolds were rinsed three times with PBS. To keep the cells hydrated, 50 μl of PBS was added to each well. Finally, cells were observed using a fluorescence microscope with an excitation wavelength of 358 nm and emission at 461 nm.

### Cell migration test

The Wound Scratch Test, also known as the Scratch Assay or Wound Healing Assay, is a method used to study cell migration. In this test, a scratch or wound is created on a cultured cell layer using a sterile instrument such as a pipette tip or a specialized scraper. The cells are then allowed to migrate and gradually close the wounded area. Images are taken at specific time points, and the reduction in the scratched area is analyzed to assess the extent of wound closure. To achieve complete growth, 3T3 cells were seeded into 96-well plates and incubated. After gently scratching the confluent cell monolayers with a pipette tip, the debris was removed by washing with medium. Migration was documented using an Inverted Leica Fluorescence Microscope at 12, 24, and 48 hr of culture. The distance covered by scratch closure was measured to quantify the extent of cell migration (formula provided below).

Healing (%) = Area of original wound - Area of wound during healing / Area of original wound × 100

### Surgical protocol

Animal experiments conducted in accordance with ethical standards were approved by the ethics committee of Shahroud Medical University (IR.SHMU.REC.). Six healthy adult male Wistar rats, each weighing between 200 and 220 grams, were anesthetized via intraperitoneal injection of xylazine (10 mg/kg) and ketamine (100 mg/kg). After anesthesia, a full-thickness wound measuring 15 × 15 mm² was created at a predetermined site. The six rats were divided into six groups for this study, with each group receiving different treatments, including a negative control, a positive control, and a treatment group receiving a sample containing GO nanoparticles and carbomer polymer. The samples were applied to the wound area and secured with elastic adhesive bandages and sterile gauze. Wound healing progress was evaluated 14 days post-surgery by capturing wound images with a stationary digital camera (Canon Inc., Tokyo, Japan) set against a 10-cm scale to trace wound margins. The percentage of wound closure was measured at days 7, 10, and 14 using ImageJ software to analyze the reduction in wound area. The percentage of wound closure was then calculated using the following formula.

Cn (%) = A0 - An / A0 × 100

Where Cn is the percentage of wound area reduction at d7, d10, and d14 post-wounding, A0 is the original wound area, and An represents the wound area at d7, d10, and d14 post-wounding.

### Hematoxylin-eosin (H&E)

The H&E staining technique is commonly used in histological studies to examine and assess various body tissues. Skin samples were collected and fixed in 10% formalin (pH 7.26) fourteen days after the animals were anesthetized with ketamine (200 mg/kg body weight) and xylazine (20 mg/kg body weight). After 48 hr, the tissues were sectioned into 5μm thick slices and stained using H&E and VVG methods. An unbiased evaluator then analyzed epithelialization, angiogenesis, fibroplasia, and granulation tissue formation across the different groups using a light microscope (BX51; Olympus, Tokyo, Japan) equipped with a digital camera (DP72; Olympus) at magnifications of 40x, 100x, and 400x.

### Animal ethics statement

All animal studies and biological evaluations in this research were carried out strictly following the ARRIVE guidelines, the U.K. Animals (Scientific Procedures) Act of 1986, the EU Directive 2010/63/EU regulating animal experiments, and the National Institutes of Health’s Guide for the Care and Use of Laboratory Animals (NIH Publications No. 8023, revised 1978). The study’s experimental protocol received approval from the Shahroud University of Medical Sciences ethics committee (Approval Number: IR.SHMU.REC.1401.166).

### Statistical analysis

Quantitative results were expressed as the mean value from at least triplicate samples ± standard deviation. Statistical analysis was performed using one-way ANOVA and Student’s t-test (SPSS software 13.0, USA; GraphPad Prism 7.0, USA). A value of *P*<0.05 was considered to be statistically significant.

## Results

### SEM results

**Figure 1:**
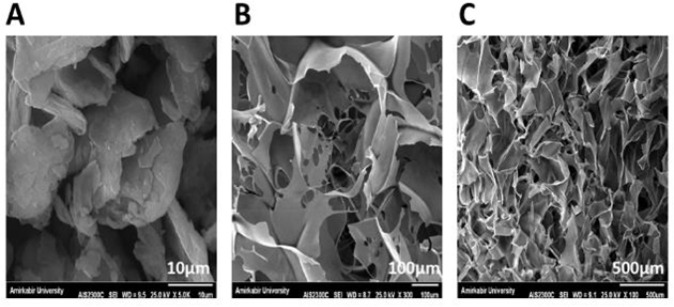
Electron microscope images showing

SEM images of GO NPs (Figure 1A) show a nanosheet structure with a smooth and uniform surface. In these images, graphene is usually seen as thin layers or an arrangement of sheets with a relatively uniform distribution on the surface. However, it is possible to observe some areas of weakly bonded graphene or surface damage, as graphene can be brittle under certain conditions. SEM images of the carbomer 980 hydrogel (Figure 1B) show a porous and networked structure with small pores and large pores. The measurements showed that the pore size of the carbomer 980 hydrogel was 72.56 ± 33.91 μm, indicating a porous structure with a variety of sizes in the carbomer structure. Examination of the images of the carbomer 980 and GO blend indicates a uniform dispersion of GO sheets in the carbomer 980 hydrogel structure, such that the GO NPs sheets are dispersed or layered in the carbomer network. This blend may represent a porous and networked structure with increased surface homogeneity and enhanced mechanical properties (Figure 1C).

### FTIR results

**Figure 2:**
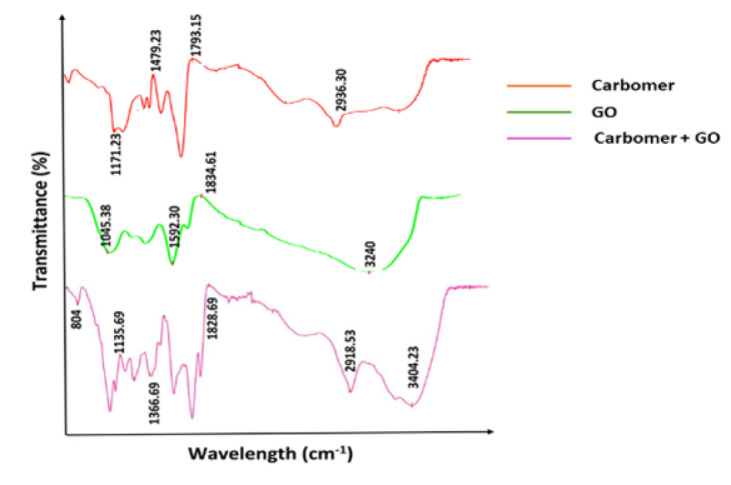
Fourier transform infrared spectroscopy (FT-IR) spectra of the studied samples showing characteristic absorption bands corresponding to functional groups in each material

Figure 2 shows the FTIR spectra of the studied groups. The red spectrum shows characteristic peaks of carbomer hydrogel. The peak observed at 1171.23 cm^-1^ indicates C-O stretching vibrations in carboxylate groups, the peak at 1479.23 cm^-1^ indicates C-H bending vibrations in carboxylate groups, the peak at 1793.15 cm^-1^ indicates C=O stretching vibrations in carboxylate groups, and the peak at 2936.30 cm^-1^ indicates C-H stretching vibrations of methylene (CH_2_) and methyl (CH_3_) groups. The green spectrum shows the characteristic peaks of GO. In this spectrum, the peak at 1045.38 cm^-1^ represents the stretching vibrations of the C-O-C bond in ether groups, the peak at 1592.30 cm^-1^ represents the stretching vibrations of C=C, the peak at 1834 cm^-1^ represents the stretching vibrations of C=O in carbonyl groups, and the peak at 3240 cm^-1^ represents the stretching vibrations of O-H in hydroxyl groups. The purple spectrum shows characteristic peaks of carbomer in combination with GO. The peak at 804 cm^-1^ represents the C-H bending vibrations in alkyl groups, the peak at 1135.69 cm^-1^ represents the C-O stretching vibrations, the peak at 1366.69 cm^-1^ represents the C-C stretching vibrations, the peak at 1828.69 cm^-1^ represents the C=O stretching vibrations, the peak at 2918.53 cm^-1^ represents the C-H stretching vibrations, and the peak at 3404.23 cm^-1^ represents the O-H stretching vibrations in hydroxyl groups.

### MTT test

**Figure 3:**
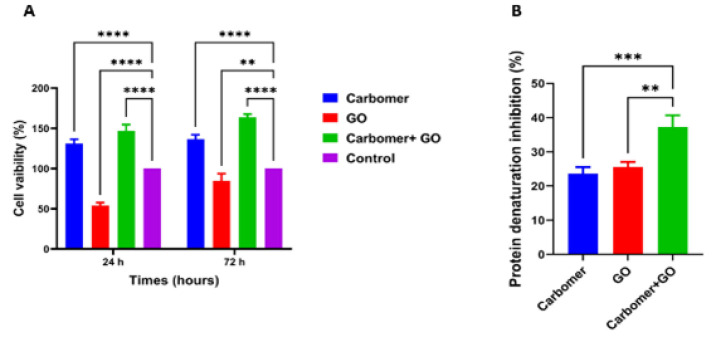
(A) Viability of 3T3 fibroblast cells under incubation with Carbomer 980, GO, and carbomer + GO. Measured by the indirect MTT assay. (B) Investigation of the degree of inhibition of protein degradation in the hydrogel designed using carbomer 980 and GO NPs

In this study, the proliferation and viability of 3T3 cells were assessed using the MTT assay at 24 and 72 hr of culture. As shown in Figure 3 (A), the cells treated with the combination of carbomer 980 and GO NPs exhibited a significantly higher survival rate at both time points compared to cells treated with either carbomer 980 or GO NPs alone, as well as the control group. Additionally, treatment with GO NPs alone resulted in a notable decrease in cell viability relative to the control. Conversely, cells treated with carbomer 980 hydrogel demonstrated a significant increase in survival rate compared to the control group.

The improved cell viability with the combined treatment likely results from the carbomer 980 hydrogel providing a supportive, biocompatible environment that enhances cell attachment and reduces toxicity. The hydrogel may also regulate the release of GO NPs, diminishing their potential to induce oxidative stress. Meanwhile, GO NPs alone can decrease cell viability due to their capacity to generate ROS and cause cellular damage. Overall, the hydrogel mitigates GO NPs’ toxicity and promotes cell survival, which explains the observed effects.

### Anti-inflammatory test results

This study explored the anti-inflammatory effects of three groups: carbomer 980, GO NPs, and the combination of carbomer 980 with GO NPs. Based on the results presented in Figure 3 (B), carbomer 980 alone showed a weak anti-inflammatory effect and had a significant decrease compared to the third group. GO NPs also have a higher anti-inflammatory effect than carbomer 980. In addition, the third group, the combination of carbomer and GO NPs, showed the highest anti-inflammatory effect due to the interaction between these two and had a significant increase compared to carbomer 980 and GO NPs.

The findings indicate that while carbomer 980 alone exhibits a modest anti-inflammatory effect, it is significantly less effective compared to the combined treatment with GO NPs. The observed increase in anti-inflammatory activity in the third group suggests a synergistic interaction between carbomer 980 and GO NPs. This enhanced effect may be attributed to the distinct properties of GO NPs, which are known for their ability to modulate inflammatory pathways, possibly through the scavenging of ROS or modulation of cytokine expression. The increased anti-inflammatory effect in the combination group indicates a synergistic interaction between the hydrogel and GO NPs, enhancing their individual benefits. This synergy could allow for lower doses and fewer side effects, highlighting the potential of the carbomer-GO NP composite as an effective anti-inflammatory treatment and encouraging further research into its mechanisms and *in vivo* applications.

### LDH test

**Figure 4:**
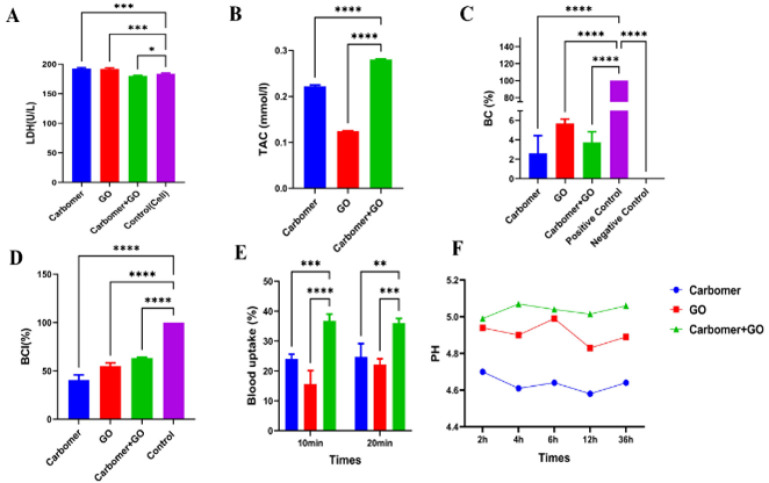
(A) LDH test of the designed hydrogel using carbomer 980 polymer containing GO NPs. (B) Comparison of total anti-oxidant capacity (TAC) in different groups. Evaluation of the rate of hemolysis of red blood cells and the blood coagulation index of the hydrogel designed based on carbomer 980 containing GO NPs. (C) Percentage of hemolysis of red blood cells: The combination of carbomer 980 and GO NPs reduces hemolysis of red blood cells. (D) BCI index of the hydrogel synthesized based on carbomer 980 polymer and GO NPs. (E) Blood absorption test results. Carbomer 980 shows the highest absorption, while the combination of carbomer 980 with GO NPs has the highest blood absorption among the samples. (F) Evaluation of pH changes of the studied groups at time intervals of 2, 4, 6, 12, and 36 hr. (****P<0.0001). GO NPs: Graphene oxide nanoparticles

For this study, LDH levels were measured as an indicator of cytotoxicity. Based on the results obtained from Figure 4A, the amount of LDH released from the cells in the first and second groups did not differ significantly from each other. However, it increased significantly compared to the control group. However, in the third group, i.e., the combination of carbomer 980 and GO NPs, this level decreased significantly compared to the other groups, which actually indicates a lower level of cytotoxicity of this group compared to the other groups.

Our LDH assay results match the MTT findings, as both tests indicate reduced cytotoxicity in the group treated with the combination of carbomer 980 and GO NPs. The decreased LDH release suggests that this combination minimizes cell membrane damage, thereby preserving cell integrity. Conversely, the elevated LDH levels in the other groups reflect higher cellular injury, consistent with their lower viability observed in the MTT assay. Together, these findings demonstrate that the synergistic effect of carbomer 980 and GO NPs not only enhances cell viability but also reduces cytotoxic effects, supporting their potential safety and efficacy for biomedical applications.

### TAC test

In this study, the total anti-oxidant capacity test results (Figure 4B) indicate that the carbomer 980 polymer has a higher anti-oxidant capacity than the GO NPs, which may be due to the inherent properties of carbomer 980 in absorbing or neutralizing free radicals. However, the third group exhibits the highest anti-oxidant capacity and demonstrates a significant increase compared to the other two groups, indicating a synergistic effect between carbomer 980 and GO NPs.

### Blood compatibility

Analysis of the blood compatibility test results (Figure 4C) shows that carbomer exhibits a significant reduction in adverse blood reactions compared to the control group and other groups, having the lowest rate of such reactions. In contrast, GO NPs display the highest rate of adverse reactions, with a significant increase compared to the first and third groups, but a significant decrease compared to the positive control group. Additionally, combining carbomer 980 with GO NPs shows blood compatibility levels between those of the first and second groups. It demonstrates a significant decrease compared to the positive control group. Overall, all groups exhibit acceptable blood compatibility (below 8%).

### BCI index

Based on the BCI test (Figure 4D), which is considered an index of blood compatibility, carbomer 980 shows the highest level of blood compatibility, with a significant decrease compared to the control group and other groups. Additionally, significant decreases are also observed in the second and third groups compared to the control group.

### Blood uptake test

In the blood absorption test results (Figure 4E), it was found that both carbomer 980 and GO NPs exhibited a significant decrease in blood absorption compared to the third group. Additionally, the combination of carbomer 980 with GO NPs showed the highest blood absorption among the examined samples. This increase in absorption in the combination group is attributed to stronger interactions and enhanced mechanical and chemical properties resulting from the presence of GO NPs, which create a more suitable environment for fluid absorption.

### Evaluation of pH changes

The pH changes of the studied groups at time intervals of 2, 4, 6, 12, and 36 hr are presented in Figure 4F. Overall, only slight variations in pH are observed within these three groups, and the pH can be considered approximately constant throughout the indicated time intervals.

### Measuring water absorption

**Figure 5:**
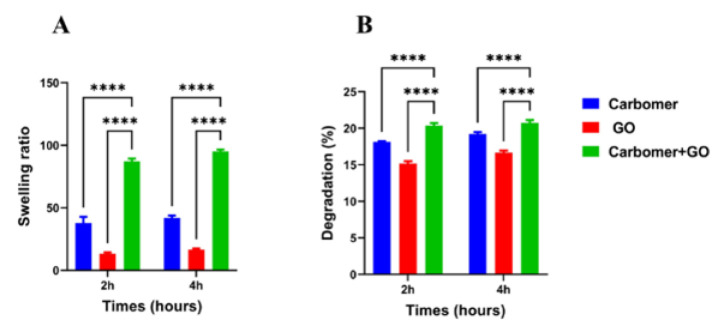
(A) Water absorption of carbomer 980 hydrogels containing GO NPs at two and four hours. Measurements show that the combination of carbomer 980 and GO NPs increases water absorption. (B) Degradability and degradation rate of carbomer 980 hydrogels containing GO NPs, indicating a higher degradation rate of the combination of carbomer 980 and GO NPs (****P<0.0001). GO NPs: Graphene oxide nanoparticles

The water and fluid absorption property is one of the most important characteristics a hydrogel should possess for effective wound healing, as it enhances cellular interactions. In this study, the water absorption rate was investigated at time intervals of two and four hours. Analysis of the results (Figure 5A) showed that the combination of carbomer 980 and GO NPs significantly increased the water absorption capacity of the synthesized hydrogel compared to the first and second groups at both time points. This enhancement is attributed to carbomer’s inherent high water absorption capacity. Additionally, graphene provides a larger surface area for water absorption due to its unique structure. The combination forms a three-dimensional network that more effectively retains water molecules. Furthermore, graphene acts as a structural enhancer by increasing the hydrophilic properties of carbomer, thereby promoting greater water absorption in the composite hydrogel (21).

### Measuring the rate of degradation

The percentage weight loss of hydrogels containing GO NPs at two and four hours is summarized in Figure 5B. Data analysis reveals that the degradation rate of the hydrogel containing GO NPs (group 3) is higher than that of the other groups, showing a significant increase compared to groups 1 and 2. This enhanced degradation is attributed to graphene’s ability to create microscopic spaces within the carbomer structure, which facilitates the absorption and subsequent release of water or other molecules. Additionally, graphene may influence the thermodynamic properties of the composite, promoting the escape of volatiles such as water from the hydrogel matrix. These factors collectively contribute to the observed weight loss of the compound over time.

### Results of the DAPI test

**Figure 6:**
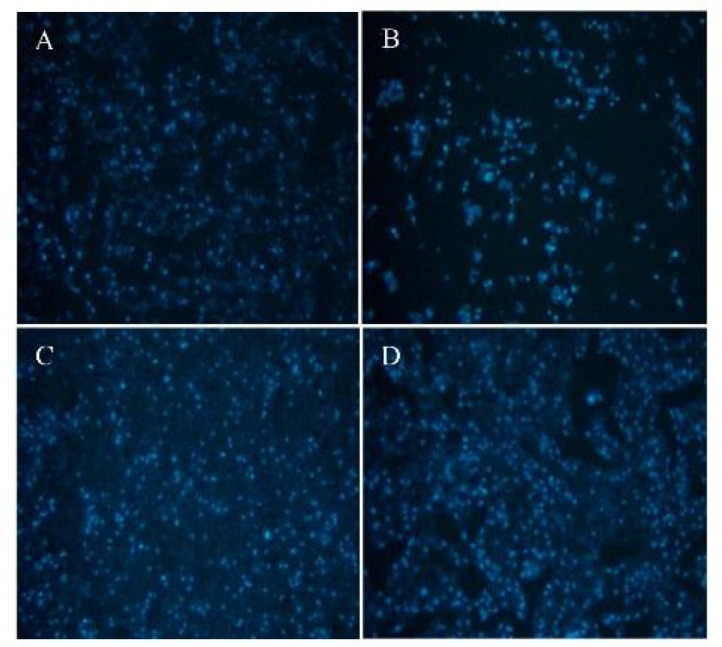
DAPI test results of the study groups

For this study, DAPI staining was used to assess cell health and nucleus count across different groups. The results (Figure 6) showed that both carbomer 980 and GO NPs alone effectively maintained cell health and reduced cell death compared to the control group. Moreover, the third group, combining carbomer 980 with GO NPs, demonstrated the best outcomes, exhibiting the highest number of healthy cells.

### Results of the in vitro cell migration

**Figure 7:**
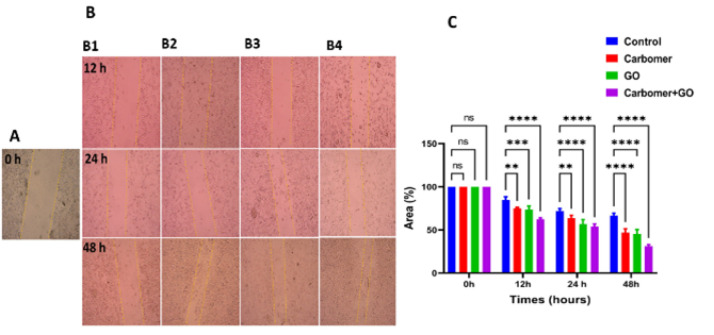
Wound scratch test for cell migration study

The results of the cell migration experiment are presented in Figure 7. A significant reduction in the scratched area was observed in cells treated with carbomer 980 hydrogel containing GO NPs at time intervals of 12, 24, and 48 hr compared to the control group.

### In vivo study

**Figure 8:**
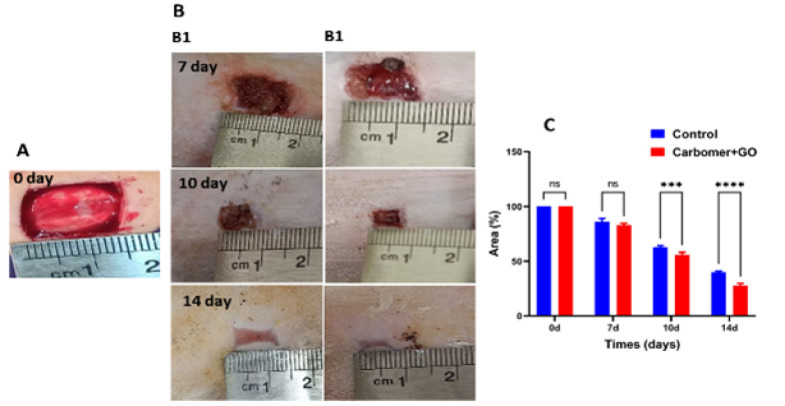
*In vivo *experiments for evaluation of wound closure

Macroscopic images of wound healing are presented in Figures 8A and 8B. Visual examination of the images reveals a significant improvement in wound healing in the group treated with carbomer 980 polymer containing GO NPs after 14 days of treatment. Notably, no signs or symptoms of infection were observed in any of the groups. To evaluate the progress of wound healing and the reduction in wound size, the rate of wound closure was measured (Figure 8C). The results indicate that the group treated with carbomer 980 polymer containing GO NPs exhibited significantly better healing than the control group.

### Hematoxylin-eosin (H&E) staining results

**Figure 9:**
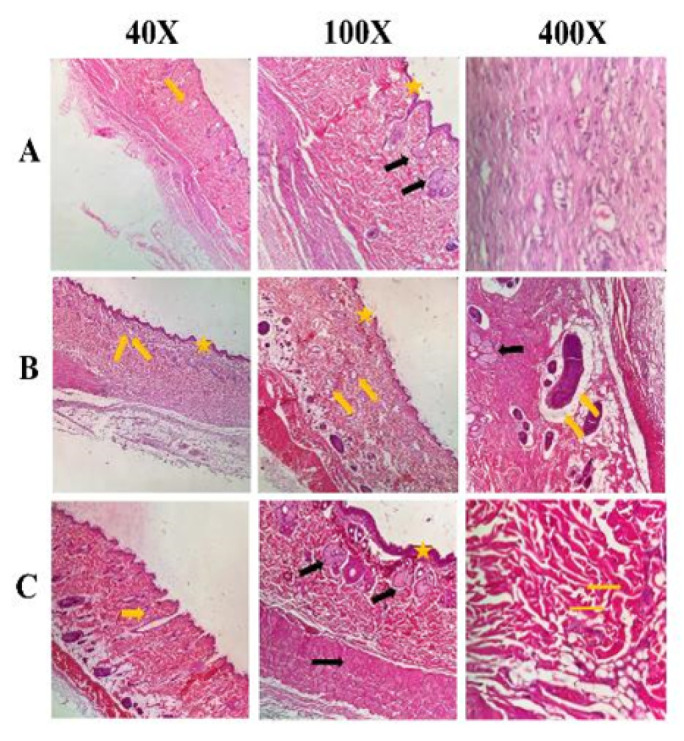
Review of H & E staining results of rat wound tissue

After treating mice with the optimal group, i.e., the combination of carbomer 980 and GO NPs for 14 days and storing tissue samples in 10% formalin, the tissue slides were stained and then examined. The results showed that skin regeneration occurred within 14 days. The results of H&E staining (Figure 9) demonstrated that in the group treated with carbomer 980 containing GO NPs, blood vessel formation and epidermis regeneration were greater than in the other groups. Additionally, the formation of hair follicles, normal epidermis, and sebaceous glands was maximized in this group. Furthermore, the amount of collagen synthesis was higher in this group compared to the others.

## Discussion

Graphene, due to its two-dimensional nanoparticle form, distinctive architecture, excellent mechanical strength, and broad, smooth surface, significantly contributes to enhancing wound healing (22, 23). Its planar structure offers an effective base for cell attachment. It supports cell interaction through electrostatic and van der Waals forces, both of which are essential for cellular growth and multiplication. Additionally, graphene accelerates wound repair by activating angiogenesis pathways and boosting nitric oxide levels (24). These attributes position graphene as a valuable material in wound management and tissue engineering. In contrast, carbomer—a hydrogel polymer known for its high water absorption—helps preserve a moist and protective setting for cells, ensuring optimal conditions necessary for their growth and survival. This moisture retention is particularly vital in promoting faster wound recovery. The synergistic combination of graphene and carbomer offers improved conditions for tissue regeneration and wound healing. While carbomer helps sustain a moist environment that shields cells from mechanical injury, graphene contributes its superior mechanical and surface characteristics to boost cell attachment and multiplication. Findings from MTT, DAPI, and wound scratch assays revealed that this composite notably increases cell survival and growth, demonstrating its excellent biocompatibility.

In addition, combining graphene with carbomer enhances blood compatibility and minimizes harm to blood cells. While pure graphene’s sharp and rigid surface may damage cell membranes, carbomer’s ability to create a moist and protective layer helps lessen these adverse effects, reducing the risk of clotting and hemolysis. This benefit is especially valuable for medical applications where materials are in direct contact with blood cells. The hydrophilic quality of carbomer is further improved by graphene’s extensive surface area, which increases water adsorption; as a result, the composite’s three-dimensional network holds water more effectively, offering a more stable environment for cells. Furthermore, the various functional groups on GO allow it to bind growth factors and drugs via electrostatic interactions, thereby enhancing therapeutic outcomes (25).

With regard to anti-oxidant activity, the graphene-carbomer combination performed well in the Total Anti-oxidant Capacity (TAC) test. The large surface area and unique electronic characteristics of graphene allow it to capture and neutralize free radicals efficiently. At the same time, carbomer’s ability to maintain a moist environment enhances the stability and effectiveness of this anti-oxidant property. Lower levels of LDH observed in cultures containing the graphene-carbomer composite suggest that cell membrane damage is reduced. This is likely due to graphene improving membrane adhesion and integrity, while carbomer’s anti-inflammatory and supportive qualities further boost cell survival. FTIR analysis also indicated that the original structures of both materials were preserved and that there were positive interactions between graphene and carbomer.

. Additionally, when applied to deep wounds (1.5 × 1.5 cm), this combination led to greater collagen production, increased blood vessel formation, thicker epidermal layers, and stimulated hair follicle development. These results align with previous research and highlight the significant potential of the graphene-carbomer composite in tissue engineering and promoting faster wound healing. Overall, thanks to their complementary and synergistic properties—improving cell compatibility, maintaining an optimal environment, and advancing tissue repair—this composite emerges as a promising and practical candidate for wound dressings and biological scaffolds (26-31).

(A) GO NPs with typical sheet-like morphology, (B) Carbomer 980 polymer showing a gel-like structure, and (C) Combination of carbomer 980 and GO NPs, illustrating the uniform dispersion of nanoparticles within the polymer matrix

GO NPs: Graphene oxide nanoparticles

The combination of these two materials leads to inhibition of protein degradation (****P<0.0001). GO: Graphene oxidena noparticles; MTT: 3-(4,5-dimethylthiazol-2-yl)-2,5-diphenyltetrazolium bromide

(A) Control group. (B) Carbomer 980. (C) GO NPs. (D) Combination of carbomer 980 polymer and GO NPs (40X). GO NPs: Graphene oxide nanoparticles; DAPI: 4′,6-diamidino-2-phenylindole

(A) Control group. (B) Cell migration results of the studied groups at 12, 24, and 48 hr ((B1) Control, (B2) Carbomer 980, (B3) GO, and (B4) Carbomer 980 +GO). (C) Cell migration experiment diagram of the studied groups (40X) ****P<0.0001). GO NPs: Graphene oxide nanoparticles

Macroscopic images of (A) control group and (B) healing wound after 7, 10, and 14 days ((B1) Control, (B2) Carbomer + GO). (C) Diagram of wound closure ) ****P<0.0001). GO: Graphene oxide nanoparticles

A: Negative control group. B: Positive control group and C: Group treated with carbomer 980 hydrogel containing GO NPs at 40X, 100X, and 400X magnification. Blue star indicates dead skin, yellow star indicates epithelium, wide yellow arrow indicates hair follicle, wide black arrow indicates sebaceous glands, narrow black arrow indicates angiogenesis, and yellow arrow indicates mature collagen.

## Conclusion

The results of this study show that the combination of carbomer 980 and GO NPs in hydrogel systems can effectively accelerate the repair and regeneration processes of tissues. This combination increases collagen formation and blood vessel growth (angiogenesis), improves epidermal thickness, and stimulates hair follicle growth. Also, the anti-oxidant effects and reduction of oxidative stress in the combination of these two materials help improve cellular health and reduce tissue damage. In general, the use of the combination of carbomer 980 and GO NPs as a novel solution in wound healing and regeneration of damaged tissues can have high potential for clinical applications in the treatment of wounds and skin disorders.

## Declaration

We acknowledge the utilization of ChatGPT for linguistic correction purposes.

## References

[1] Xu C, Akakuru OU, Ma X, Zheng J, Zheng J, Wu A (2020). Nanoparticle-based wound dressing: recent progress in the detection and therapy of bacterial infections. Bioconjug Chem.

[2] Zhou S, Xie M, Su J, Cai B, Li J, Zhang K (2023). New insights into balancing wound healing and scarless skin repair. J Tissue Eng.

[3] Mirhaj M, Labbaf S, Tavakoli M, Seifalian AM (2022). Emerging treatment strategies in wound care. J Interv Wound Care.

[4] Shi C, Wang C, Liu H, Li Q, Li R, Zhang Y (2020). Selection of appropriate wound dressing for various wounds. Front Bioeng Biotechnol.

[5] Randviir EP, Brownson DA, Banks CE (2014). A decade of graphene research: Production, applications and outlook. Microchim Acta.

[6] Zhen Z, Zhu H (2018). Structure and properties of graphene. Graphene.

[7] Shirshahi V, Tabatabaei SN, Hatamie S, Saber R (2019). Functionalized reduced graphene oxide as a lateral flow immunoassay label for one-step detection of Escherichia coli O157:H7. J Pharm Anal.

[8] Rehman SR, Augustine R, Zahid AA, Ahmed R, Tariq M, Hasan A (2019). Reduced graphene oxide incorporated GelMA hydrogel promotes angiogenesis for wound healing applications. Int J Nanomedicine.

[9] Shariati A, Hosseini SM, Chegini Z, Seifalian A, Arabestani MR (2023). Graphene-based materials for inhibition of wound infection and accelerating wound healing. J Biomed Pharm Ther.

[10] Saedi M, Shirshahi V, Mirzaii M, Nikbakht M (2024). Preparation of graphene oxide nanoparticles and their derivatives: Evaluation of their antimicrobial and anti-proliferative activity against 3T3 cell line. J Dent Sci Technol.

[11] Shirshahi V, Saedi M, Nikbakht M, Mirzaii M (2023). Unveiling the antimicrobial potential of oxidized graphene derivatives: Promising materials for advanced wound dressings and antibacterial surfaces. J Dent Dent Sci Technol.

[12] Lasocka I, Jastrzębska E, Szulc-Dąbrowska L, Skibniewski M, Pasternak I, Kalbacova M (2019). The effects of graphene and mesenchymal stem cells in cutaneous wound healing and their putative action mechanism. Int J Nanomedicine.

[13] Li Z, Wang H, Yang B, Sun Y, Huo R (2015). Three-dimensional graphene foams loaded with bone marrow derived mesenchymal stem cells promote skin wound healing with reduced scarring. Mater Sci Eng C.

[14] Manral K (2015). Viscoelastic properties and rheological characterization of carbomers. Int J Res Eng Technol.

[15] Hui Q, Zhang L, Yang X, Yu B, Huang Z, Pang S (2018). Higher biostability of rh-aFGF-carbomer 940 hydrogel and its effect on wound healing in a diabetic rat model. J Drug Deliv Technol.

[16] Singla AK, Chawla M, Singh A (2000). Potential applications of carbomer in oral mucoadhesive controlled drug delivery system: a review. Drug Dev Ind Pharm.

[17] Huang Y, Shi F, Wang L, Yang Y, Khan BM, Cheong K-L (2019). Preparation and evaluation of Bletilla striata polysaccharide/carboxymethyl chitosan/carbomer 940 hydrogel for wound healing. Int J Biol Macromol.

[18] Hayati F, Ghamsari SM, Dehghan MM, Oryan A (2018). Effects of carbomer 940 hydrogel on burn wounds: An in vitro and in vivo study. J Drug Deliv Technol.

[19] Patarroyo JL, Cifuentes J, Muñoz LN, Cruz JC, Reyes LH (2022). Novel antibacterial hydrogels based on gelatin/polyvinyl-alcohol and graphene oxide/silver nanoconjugates: Formulation, characterization, and preliminary biocompatibility evaluation. J Health.

[20] Chen C-Y, Yin H, Chen X, Chen T-H, Liu H-M, Rao S-S (2020). Ångstrom-scale silver particle–embedded carbomer gel promotes wound healing by inhibiting bacterial colonization and inflammation. Adv Healthc Mater.

[21] Fu C, Qi Z, Zhao C, Kong W, Li H, Guo W (2021). Enhanced wound repair ability of arginine-chitosan nanocomposite membrane through the antimicrobial peptides-loaded polydopamine-modified graphene oxide. Int J Biol Macromol.

[22] Priyadarsini S, Mohanty S, Mukherjee S, Basu S, Mishra M (2018). Graphene and graphene oxide as nanomaterials for medicine and biology application. J Nanomater Chem.

[23] Kumara P, Prakash S, Lokesh P, Manral K (2015). Viscoelastic properties and rheological characterization of carbomers. Int J Life Res Eng Technol.

[24] Mukherjee S, Sriram P, Barui AK, Nethi SK, Veeriah V, Chatterjee S (2015). Graphene oxides show angiogenic properties. J Biomed Nanotechnol.

[25] Huang X, Yang J, Zhang R, Ye L, Li M, Chen W (2022). Phloroglucinol derivative carbomer hydrogel accelerates MRSA-infected wounds’ healing. Int J Mol Sci.

[26] Wang Z, Hu Y, Xue Y, Zhu Z, Wu Y, Zeng Q (2022). Log P determines licorice flavonoids release behaviors and classification from carbomer cross-linked hydrogel. Int J Mol Sci.

[27] Sari LORK, Fardliana LQ, Nurahmanto D, Irawan ED (2023). Carbomer and ethyl cellulose optimisation in the preparation of mucoadhesive microspheres ciprofloxacin hydrochloride. J Pharm Educ.

[28] Gan C, Cheng R, Xu K, Zhang J, Wang H, Xu T (2023). Preparation and physicochemical properties of coenzyme Q10 loaded niosomal hydrogels based on carbomer and scleroglucan. Int J Pharm.

[29] Dehghanzad B, Aghjeh MKR, Rafeie O, Tavakoli A, Oskooie A (2016). Synthesis and characterization of graphene and functionalized graphene via chemical and thermal treatment methods. RSC Adv.

[30] Hosseini MA, Malekie S, Ebrahimi N (2020). The analysis of linear dose-responses in gamma-irradiated graphene oxide: Can FTIR analysis be considered a novel approach to examining the linear dose-responses in carbon nanostructures?. RSC Adv.

[31] Zhang L, Li Y, Guo H, Zhang H, Zhang N, Hayat T (2019). Decontamination of U (VI) on graphene oxide/Al₂O₃ composites investigated by XRD, FT-IR and XPS techniques. J Hazard Mater.

